# Differential Cellular Responses to Hedgehog Signalling in Vertebrates—What is the Role of Competence?

**DOI:** 10.3390/jdb4040036

**Published:** 2016-12-10

**Authors:** Clemens Kiecker, Anthony Graham, Malcolm Logan

**Affiliations:** 1 Department of Developmental Neurobiology, King’s College London, Hodgkin Building, Guy’s Hospital Campus, London SE1 1UL, UK; anthony.graham@kcl.ac.uk; 2 Randall Division of Cell & Molecular Biophysics, King’s College London, Hodgkin Building, Guy’s Hospital Campus, London SE1 1UL, UK; malcolm.logan@kcl.ac.uk

**Keywords:** chick embryo, *Drosophila*, iroquois, IRX, limb bud, morphogen, mouse, neural tube, proliferation, temporal adaptation, zebrafish

## Abstract

A surprisingly small number of signalling pathways generate a plethora of cellular responses ranging from the acquisition of multiple cell fates to proliferation, differentiation, morphogenesis and cell death. These diverse responses may be due to the dose-dependent activities of signalling factors, or to intrinsic differences in the response of cells to a given signal—a phenomenon called *differential cellular competence*. In this review, we focus on temporal and spatial differences in competence for Hedgehog (HH) signalling, a signalling pathway that is reiteratively employed in embryos and adult organisms. We discuss the upstream signals and mechanisms that may establish differential competence for HHs in a range of different tissues. We argue that the changing competence for HH signalling provides a four-dimensional framework for the interpretation of the signal that is essential for the emergence of functional anatomy. A number of diseases—including several types of cancer—are caused by malfunctions of the HH pathway. A better understanding of what provides differential competence for this signal may reveal HH-related disease mechanisms and equip us with more specific tools to manipulate HH signalling in the clinic.

## 1. Introduction

Classical embryologists postulated over 100 years ago that cells in a developing multicellular organism communicate with each other: some cells emit signals, *inducers*, that elicit specific changes in the receiving target cells. Around that time it was also recognised that the target cells need to be able to interpret the signal—they need to be *competent* [1,2]. Over the last 30 years, the rise of molecular biology has led to the identification of the signals that induce a multitude of cellular behaviours and identity changes during development. One of the biggest surprises from this research was that a small number of signalling pathways (less than a dozen) seem to direct not only the formation of the hundreds of different cell types that make up a functional organism, but they also regulate cellular proliferation, morphogenesis, motility and cell death. Moreover, the same signals are also active in adult homeostasis, and consequentially their deregulation can cause diseases such as cancer.

**Figure 1:**
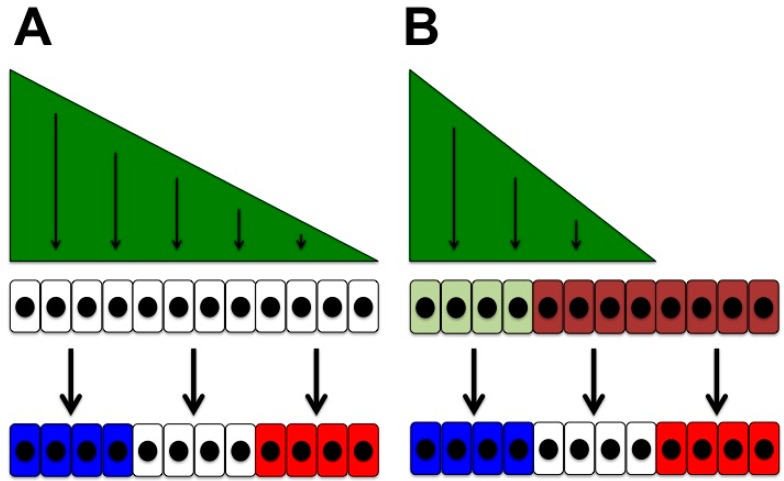
Two scenarios for the induction of multiple cellular responses (blue, white, red) by a signalling factor (green). (**A**) The factor acts as a *morphogen* that induces multiple responses dose-dependently; (**B**) A pre-pattern in the receiving cells (light green vs. brown) results in differential responses to the signal—the receiving cells display *differential competence*.

This raises the question of how a small number of signals can generate such a broad range of cellular responses. There are two—not necessarily mutually exclusive—possible answers to this question:

1. Inducing signals may elicit multiple cellular responses in a dose-dependent fashion, i.e., they function as morphogens (Figure 1A) [3].
2. Cellular responses may depend on cell-intrinsic factors that reflect the context of the responding cell, its location and developmental history (Figure 1B). This contextual ability to respond to a signal brings us back to the idea of cellular competence: cells that respond differently to a given signal display *differential competence* for this signal.

In the first scenario, differential cellular responses are determined by different doses of the inducer, a non cell-autonomous factor. In contrast, differential competence can be defined as a property inherent to the responding cell that determines how this cell responds to a given signal. Biologists have made tremendous progress in establishing the molecular mechanics of the signalling pathways that underlie cell-to-cell communication, but our understanding of what mediates differential competence is lagging behind.

In this review, we focus on the phenomenon of differential competence with regards to the Hedgehog (HH) pathway—a signalling cascade that is found across the animal kingdom, that is involved in multiple processes during embryogenesis as well as adult homeostasis, and that is frequently defective in different types of cancers and other diseases. We begin by providing a brief outline of the HH pathway and some examples for its various roles. Dose-dependent signalling of HH has been described in several embryonic tissues, and interactions with other signalling pathways have been studied in a plethora of different scenarios. Here we will concentrate on cases where differential responses to HH signalling have been attributed to differential competence. We will discuss the factors that establish such differential competence and review what is known about the mechanisms by which they could change the response of a cell to HH.

## 2. A Brief Outline of the Hedgehog Pathway

*Hh* genes are found throughout the animal kingdom. Vertebrates have three *hh*s: *Desert hedgehog (Dhh)*, *Indian hedgehog (Ihh)* and *Sonic hedgehog (Shh)*, although *Ihh* and *Shh* have undergone duplications in some teleosts [4]. The HH ligand is bound by a large multipass transmembrane protein called Patched (PTC). In the absence of HH, PTC inhibits the signalling activity of the seven-pass transmembrane protein Smoothened (SMO); however, once PTC has bound HH, this inhibition is relieved and SMO can trigger an intracellular signalling cascade. Initial studies suggesting that PTC and SMO form a physical complex have been refuted, as PTC was shown to inhibit SMO sub-stoichiometrically, indicating that a catalytic mechanism must be at work [5,6]. The structural similarity of PTC with small molecule transporters suggests that it could function by shuttling a small regulator of SMO across the cell membrane [7,8]. It is a common assumption that all cells of early vertebrate embryos express at least one of the two *Ptc* genes and *Smo*, at least at low levels, and are therefore in principle competent to respond to HHs [9].

Other membrane-bound proteins also bind HH ligands: members of the IHOG (Interference HedgeHOG) family such as CDO (Cell adhesion molecule-related/Downregulated by Oncogenes) and BOC (Brother Of CDO) as well as the vertebrate-specific protein GAS1 (Growth Arrest-Specific gene 1) function as co-receptors that may promote the de-repressive effect of HH on PTC, or limit the range of HH by sequestering it [10,11,12,13,14]. The single-pass transmembrane protein HHIP (HH Interacting Protein) also functions as a competitive inhibitor of HH binding to PTC [15]. Activation of HH-SMO signalling results in removal of HHIP from the surface of the signal-receiving cells suggesting a feedforward mechanism in HH pathway activation [16].

In response to HH binding SMO undergoes a conformational switch [17] and translocates into the primary cilium, an antenna-like membrane-bound cytoplasmic protrusion on the apical surface of most vertebrate cells [18,19]. It is well established that this cellular organelle plays a crucial role in HH signalling [20]. In the absence of HH, PTC is found on its surface, but it is shuttled out of, whereas SMO and several other components of the HH pathway become enriched in, the primary cilium in response to HH activation [18,21,22]. The aetiology of several congenital disorders have been linked to ciliary dysfunction, and defective HH signalling is a likely contributor to the pathology of such ciliopathies [23].

Ultimately, HH signalling is mediated via DNA-binding zinc finger transcription factors (TFs) of the GLI family. Vertebrates have three GLIs of which GLI1 can only function as a transcriptional activator whereas both GLI2 and GLI3 undergo proteolytic processing from activators to repressors. However, GLI2 appears to be the main activator and GLI3 the main repressor of HH target genes [24]. The molecular link between SMO and the GLIs remains somewhat ill defined. SMO interacts with a protein complex assembled by the kinesin-related scaffolding protein KIF7 (Costal2 in *Drosophila*) that involves the protein kinase Fused and its suppressor SUFU as well as several kinases that phosphorylate SMO [25,26,27].

Besides regulating the transcription of target genes, SHH also functions as an axon guidance factor [28]. The timescale of the response of growth cones to SHH protein in culture indicates that this effect is independent of transcription and, consistently, GLIs are not involved in these responses which are mediated via phosphorylation of Src family kinases [29,30] and possibly via an intracellular increase in Ca^2+^ ions [31], thereby directly affecting the actin cytoskeleton. The effect of HH on the cytoskeleton is not limited to axons, but can also be found in various types of cultured cells where it is mediated via activation of small GTPases such as Rac1 and RhoA [32,33,34,35].

Finally, two of the HH receptors, PTC and CDO, have been shown to function as dependence receptors, i.e., they induce apoptosis in the absence of bound ligand [36,37]. For a detailed description of the mechanistic basis of HH signalling—which is beyond the scope of this review—we refer the reader to several excellent reviews that have been written on the topic [24,38,39]. A key question for the remaining part of this review is how cells select different responses upon HH exposure.

## 3. Roles of Hedgehog Signalling

*Hh* was originally identified through a mutation affecting segment polarity in the fruit fly *Drosophila* [40], and the core components of its signalling pathway were identified in a similar manner and organised according to their epistatic relationships [41]. Subsequently the pathway was also shown to affect patterning in the imaginal discs, the larval progenitors of *Drosophila*’*s* appendages (legs, wings, antennae, eyes) [42,43].

The vertebrate *hh*s were discovered in the early 1990s [44], and SHH rapidly became the centre of attention due to its potential role as a morphogen, a patterning factor that is released from tissues with polarising activity (‘*organiser*’ regions) and that is able to induce multiple cell fates in a dose-dependent fashion. This morphogenetic role of SHH is best understood in the developing central nervous system (CNS). *Shh* is expressed in the notochord, a rod of mesodermal tissue underlying the midline of the developing neural plate, and in the floor plate, the ventral-most population of cells in the neural tube that lies dorsal to the notochord in all vertebrates. Over the last 25 years, multiple studies in amphibians, chick, mouse and zebrafish have established that SHH-GLI signalling is the key inducer of ventral identity in the embryonic CNS: graded levels of SHH induce the identity of motor neurons, different types of ventral interneurons and floor plate cells in the ventral spinal cord and hindbrain. Additionally studies in chick and mouse have demonstrated that they set up an arc-shaped pattern of different progenitor domains in the ventral midbrain, and they are involved in establishing subpallial and hypothalamic identity in the ventral forebrain [45,46,47,48,49,50,51]. Because of its key role in pattering the ventral forebrain, defects in the SHH pathway cause holoprosencephaly, the most common congenital disorder of the forebrain which is characterised by cyclopia, a failure to separate the forebrain into two lateral hemispheres, and craniofacial malformations [52].

SHH also regulates cell fate specification in the developing limb where it dose-dependently regulates anteroposterior patterning and determines the orderly array of digits [53]. Strong evidence for this role comes from mutations in the SHH pathway that can cause either the formation of extra digits (pre- or postaxial polydactyly) or absence of digits in humans [54,55]. Furthermore SHH is involved in cell fate allocation in the somites (where it induces the sclerotome) [56,57], pituitary gland [58], intestinal epithelium (where amongst other things it antagonises pancreas development) [59], in the differentiation of muscle fibres [60] and in many other tissues of the developing vertebrate embryo. SHH also induces taste buds in lingual epithelium [61] and is essential for the formation of hair follicles and teeth [62,63]. DHH plays a role in male germ line development [64] and IHH controls the rate of cartilage differentiation during skeletogenesis [65].

Besides specifiying different cell fates as a morphogen, SHH also has an effect on proliferation: for example, the expansion of the neocortex, diencephalon, tectum and cerebellum depends on SHH signalling [66,67,68,69,70]. This growth-promoting function is probably mediated through transcriptional upregulation of *Cyclin D1* and *Myc*, both encoding positive regulators of the cell cycle [71,72,73]. Moreover, PTC directly interacts with Cyclin B1, sequestering it away from the nucleus and thereby blocking the completion of mitosis, and this interaction is antagonised by HH binding to PTC [74].

HH signalling affects tissue homeostasis by regulating the balance between stem cell self-renewal and differentiation. For example, both *IHH* and *SHH* are expressed in the crypts of the intestinal epithelium where they restrict the stem cell population to the base and promote their differentiation into enterocytes as they exit the crypts [75]. In the nervous system, SHH has long been known to promote proliferation of neural precursor cells [76]; however, more recently it has emerged that it is also required for the maintenance of the adult neural stem cell pool [77,78,79,80]. Moreover, SHH signalling becomes upregulated in reactive astrocytes following a local freeze injury, resulting in activation of progenitor proliferation [81], and in a stretch injury model [82]. Similarly, SHH drives the regeneration of bladder epithelium following tissue damage [83].

Given its effect on (stem) cell proliferation it does not come as a surprise that aberrant activation of the HH pathway can cause tumour formation. Basal cell carcinoma [84,85], medulloblastoma [86], meningiomas [87,88,89] and rhabdomyosarcoma [90] are all known to be caused by mutations in the HH pathway, and several other types of cancer have also been associated with elevated levels of HH signalling, either in the cancer stem cells themselves or in the tumour environment [91,92].

These different effects in various tissues of the developing and adult organism are just a sample of the many roles of HH signalling, highlighting that a diverse set of distinct cellular outcomes can result following HH pathway activation (Table 1).

## 4. Differential Competence for Hedgehog

Differential cellular responses to HH signalling may be generated by its dose-dependent effects and/or by differential competence of the signal-receiving cells (Figure 1). One of the classical paradigms for HH signalling in the vertebrate embryo is the ventral neural tube which is exposed to SHH that is released from the notochord and that induces floor plate cells, motor neurons and different types of ventral interneurons of the developing spinal cord [48]. Subsequently SHH is required for the expansion of those previously formed progenitor domains [93,94] and for the production of different types of glia [95,96,97]. A similar scenario is observed in the mouse forebrain where early SHH signalling induces and patterns the subpallium and ganglionic eminences (the precursors of the basal ganglia and of many of the neocortical interneurons), whereas later signalling drives the growth of the anterior brain, in particular that of the neocortex [98]. At least two phases of SHH activity—an early patterning and a later growth-promoting one—have also been observed in the developing ventral midbrain [51], hypothalamus [50] and limb of the mouse embryo [99].

During the patterning phase differential cellular responses are caused by dose-dependent effects of SHH in these different embryonic tissues (Figure 1A). However, the range of possible responses within each of these tissues (spinal cord: floor plate, motor neurons, interneurons; forebrain: subpallium, ganglionic eminences; limb: digit identities) is limited by their differential competence (Figure 1B). Furthermore, in each of these tissues there is a temporal change in the response of SHH-receiving cells from changing their fate to proliferating and/or differentiating.

### 4.1. Temporal Changes in Competence for Hedgehog Signalling

How is the temporal transition from patterning to proliferation regulated? Elegant studies in the embryonic spinal cord of the mouse embryo have revealed that transient exposure to the ventral SHH gradient initiates a *gene regulatory network* (GRN) in the receiving cells that stabilises progenitor fates and uncouples them from an ongoing requirement for the inducing signal [100]. This uncoupling may allow SHH to take over other roles such as the promotion of growth. Simultaneously, cells exposed to high levels of SHH become less responsive to the signal due to a combination of negative feedback circuits—an effect known as *temporal adaptation*—involving upregulation of the inhibitory receptors PTC and HHIP and transcriptional regulation of the *Gli* genes [101].

A similar negative feedback loop is active in the ventral mouse forebrain where the homeodomain transcription factor NKX2.1, a target of SHH, downregulates *GLI* expression, thereby attenuating the signal in those cells that are exposed to highest levels of SHH [102]. In fact, it appears to be a common phenomenon that those cells that express *SHH*—such as in the notochord, floor plate and zona limitans intrathalamica (ZLI; see below)—become refractory or at least less sensitive to the signal. It is possible that this mechanism has developed to prevent a ‘domino effect’ in places where SHH can induce its own expression such as the ventral neural tube [98].

The desensitisation of SHH target cells by temporal adaptation is compatible with SHH’s later role in proliferation which depends on significantly lower levels of the signal: SHH has been demonstrated to promote growth even in the dorsal neural tube of the chick embryo, outside of its range of patterning [69,103]. Taken together, progenitors in different tissues of the developing embryo switch their response to SHH signalling from that of acquiring different cell fates to that of proliferating. This change in response is at least partly due to a change in competence based on temporal adaptation, resulting in a reduced sensitivity of cells to SHH exposure.

### 4.2. Cells Movements May Accompany Temporal Competence Changes

**Figure 2:**
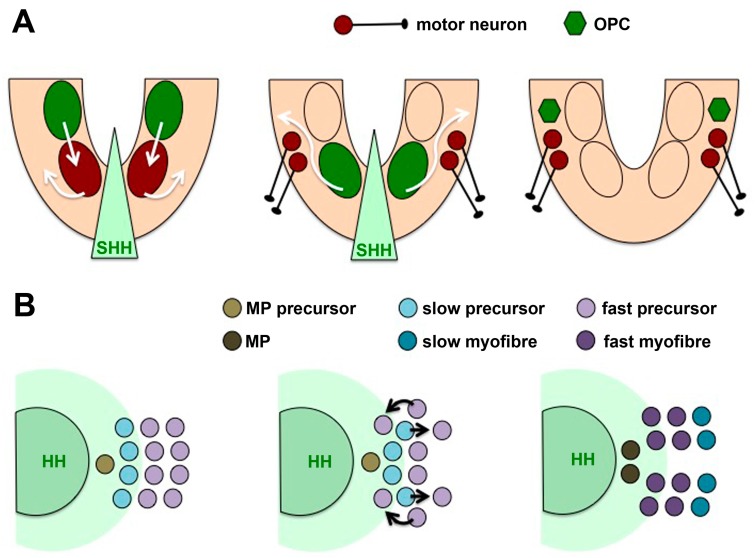
Cell movements accompanying temporal changes in competence for HH signalling. (**A**) In the ventral neural tube, progenitors in a more ventral domain (brown) are induced to become motor neurons by sonic hedgehog (SHH) (light green) from the notochord and floor plate. As these motor neurons differentiate, they move away radially and are replaced by progenitors from a more dorsal domain (dark green) which respond to SHH by differentiating into oligodendrocyte precursor cells (OPC) [104,105,106,107]; (**B**) In the zebrafish myotome, HHs from axial tissues induce muscle pioneer cells (brown) and slow-twitch myofibres (blue). After they have received the signal, the slow precursor cells (light blue) move away radially and are replaced by fast-twitch muscle precursor cells (light purple) that differentiate in response to HH [109,110].

In some cases where the temporal competence of a progenitor population for HH signalling changes, cells move away from, or into closer proximity to, the source of HH. For example, motor neurons, interneurons and oligodendrocytes are generated sequentially from *olig2*-expressing progenitors in the ventral spinal cord and hindbrain of the zebrafish embryo [104,105]. However, detailed lineage tracing analyses revealed that the progenitors giving rise to these different cell populations are not identical [106]. Motor neurons, which differentiate first, arise from more ventral regions, whereas oligodendrocytes arise from more dorsal *olig2*-positive progenitors. As the motor neurons differentiate, they radially move away from the ventricular zone, and they are replaced by glial progenitors from the more dorsal *olig2* domain [107]. Thus, differentiating motor neurons move out of the range of SHH whereas oligodendrocyte precursors move within its range, integrating temporal and spatial cell fate assignment (Figure 2A).

A similar phenomenon, although in a different type of tissue, has been observed in the zebrafish myotome, the muscle-producing part of the somite. Here HHs from the notochord initially induce slow-twitch muscle fibres [108], and the upregulation of *ptc* in those cells creates a barrier that limits the spread of HH throughout the myotome. Once they have been specified as slow-twitch progenitors and begin to differentiate, they move laterally away from the notochord, making space for another population of progenitor cells that can now receive the HH signal. Because these cells are exposed to HH at a later time point, they differentiate into fast muscle fibres (Figure 2B) [109,110].

### 4.3. Other Signalling Pathways Modulate Cellular Responses to Hedgehog Signalling

**Figure 3:**
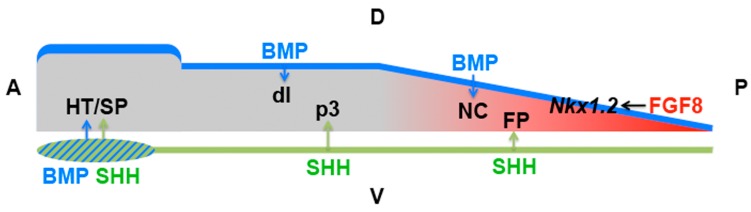
Differential competence of the elongating neural tube. Posteriorly, FGF8 (red) induces *Nkx1.2* which endows neural tube cells with competence for floor plate (FP) formation in response to SHH (green) from the notochord (and for neural crest, NC, formation in response to BMPs). More anteriorly, in the absence of *Nkx1.2*, SHH induces progenitors of ventral interneurons (p3) and BMPs (blue) induce dorsal interneurons (dI) [112]. In the forebrain, the prechordal mesendoderm expressed both BMP and SHH, resulting in the induction of the hypothalamus and subpallium (HT/SP) [120]. A, anterior; D, dorsal; P, posterior; V, ventral.

High levels of SHH signalling from the notochord induce floor plate identity at early stages of neural development whereas comparable doses induce ventral interneurons at later stages, indicating that neural progenitors lose the competence for floor plate induction [111]. A recent study in chick demonstrated that this competence is mediated by an intersection of the SHH signalling domain around the notochord with the domain of Fibroblast Growth Factor (FGF) signalling in the posterior region of the embryo (Figure 3) [112]. It has been known for a while that the mutually repressive interaction between FGF and retinoic acid signalling regulates the transition from progenitor proliferation to differentiation in the chick neural tube [113], and posterior FGF signalling seems to maintain a ‘stem zone’ during the elongation of the embryonic axis [114,115]. Using different gain- and loss-of-function approaches, Sasai et al. (2014) showed that FGF signalling is both necessary and sufficient to endow cells in the neural plate/tube with competence to form floor plate in response to high level SHH signalling. They identified the homeobox transcription factor NKX1.2 as a mediator of this effect: NKX1.2 is induced by early FGF signalling, it is necessary and sufficient to endow neural progenitors with competence to induce floor plate character in response to SHH, and it induces this competence in the absence of FGF signalling, indicating that it functions downstream of FGF (Figure 3). FGF-NKX1.2 signalling also regulates competence on the dorsal side of the neural tube in response to Bone Morphogenetic Protein (BMP) signalling—once neural cells have left the FGF domain and have ceased to express *NKX1.2*, they form dorsal interneurons instead of neural crest in response to BMPs [112]. This study provides an elegant model of how the signals that regulate dorsoventral patterning of the neural tube are integrated with anteroposterior axis elongation and the associated gradient of neural differentiation.

Whereas SHH-GLI signalling regulates pattern formation in the ventral spinal cord, BMPs and WNTs released from the roof plate and the overlying ectoderm regulate dorsal cell fate acquisition. WNT signalling is mediated by transcription factors of the T Cell Factor/Lymphoid Enhancer Factor (TCF/LEF) family. Interestingly, TCF/LEF binding sites were found in a cis-regulatory module of the SHH-inducible ventral marker *Nkx2.2*, and it appears that in the mouse embryo the dorsal limit of *Nkx2.2* expression is established through repression by TCF/LEFs, rather than threshold-dependent induction by SHH [116]. This remarkable study reveals how intersecting signalling pathways may be integrated directly at the level of transcriptional control of target genes.

Another signalling pathway that affects the competence of neural progenitors for SHH signalling is the Notch pathway. Notch signalling is well known as a regulator of neurogenesis and gliogenesis: it suppresses neural differentiation and promotes gliogenesis in both vertebrates and invertebrates [117]. In two recent studies, Kong et al. (2015) and Stasiulewicz et al. (2015) discovered that Notch signalling influences dorsoventral patterning in the ventral spinal cord by promoting ventral identity. Using gain- and loss-of-function experiments in chick and mouse as well as reporter assays in chick neural explants and in cultured cells they found that this effect is mediated via SHH pathway activation. The most striking discovery of these studies is that Notch signalling promotes both the length of the primary cilium and accumulation of SMO in the primary cilium—a hallmark of HH pathway activation. These findings indicate that Notch signalling enhances the responsiveness of cells to HH signalling, and they provide an additional explanation why undifferentiated neural progenitors (which experience high levels of Notch pathway activation) are more sensitive to SHH than differentiating neurons, yet again linking cell fate acquisition with developmental timing [118,119].

Whereas SHH induces floor plate cells, motor neurons and ventral interneurons in the spinal cord, it induces hypothalamic and subpallial cell fates in the forebrain. Work in chick embryos revealed that the prechordal mesoderm, axial mesodermal tissue that lies anterior to the notochord, transiently expresses the signalling factor BMP7 in addition to SHH, and that the combinatorial activity of these two signals results in ventral forebrain induction (Figure 3) [120]. How these two signals are integrated remains to be established.

As these studies show, other signals can modulate the cellular competence for HH signalling by functioning as upstream regulators of competence factors such as NKX1.2 (FGF signalling), TCF/LEF (WNT signalling) and SMO localisation (Notch signalling).

### 4.4. Receptor Switching Can Change the Competence for Hedgehog Signalling

The classical view of cellular competence postulated that a cell either responds to a given signal or not, and the molecular correlate of this was the presence or absence of a receptor for this signal. HHs bind to a range of different membrane-bound receptors, so it is tempting to speculate that changes of this receptor repertoire on a given cell could underlie changes in competence.

HHs can function as axon-guidance factors in a GLI-independent fashion (see above). A study in chick suggests that floor plate-derived SHH regulates the longitudinal guidance of commissural spinal axons after they have crossed the ventral midline. Intriguingly, both *PTC* and *SMO* expression have been downregulated in commissural neurons by that time, suggesting that this guidance function of SHH may be mediated by an alternative receptor. Indeed, gain- and loss-of-function experiments implicate HHIP in this process [121]. It remains to be established how HHIP—which lacks an intracellular domain—relays the signal across the axonal membrane. It is possible that in this context HHIP functions as a co-receptor that presents SHH to a hitherto unknown axon guidance receptor. Thus, switches in receptor expression may underlie some of the changes of cellular competence for HHs. Another candidate that may act as a ‘non-canonical’ axon guidance receptor is BOC which is expressed on commissural axons and has been found to be essential for the proper projection of commissural axons towards the midline in zebrafish and rodents [122,123,124].

In order to activate GLI signalling, SMO has to be localised to the primary cilium. A recent study in cell culture suggests that HH-mediated chemotaxis in different types of cultured cells does indeed depend on SMO, but that the receptor is localised outside of the cilium for this kind of signal to become activated [125]. This suggests that not only a switch of receptor types, but also a change in the localisation of a single receptor can determine the way in which a cell responds to HHs.

### 4.5. Differential Competence for HH Signalling in Different Tissue Types

The studies on neural tube and limb development discussed above indicate that the range of cellular responses to HH signalling depends on the type of responding tissue. One of the earliest and most fundamental cell fate decisions in the developing embryo is the establishment of the three germ layers: endoderm, mesoderm and ectoderm [126]. It seems likely that the first changes in competence are made alongside the formation of the germ layers.

In the ventral spinal cord (a derivative of the ectoderm) SHH induces different types of neurons whereas it patterns digit identities in the limb bud mesoderm. When studying the regulatory regions of SHH target genes in the neural tube, the Ericson lab recently found binding sites for both GLI and SOXB1 TFs in close proximity within these regulatory elements [127]. Strikingly, expression of SOXB1 factors in the developing chick limb bud resulted in the induction of a broad range of neural marker genes in response not only to SHH, but also to BMP and retinoic acid signalling [128]. Thus, neural-specific competence for a range of different signals can be switched by ectopic expression of a single transcription factor! Whether an equivalent limb mesodermal competence factor for SHH signalling exists has not yet been determined.

### 4.6. Domains of Differential Competence for HH Signalling within a Tissue

**Figure 4:**
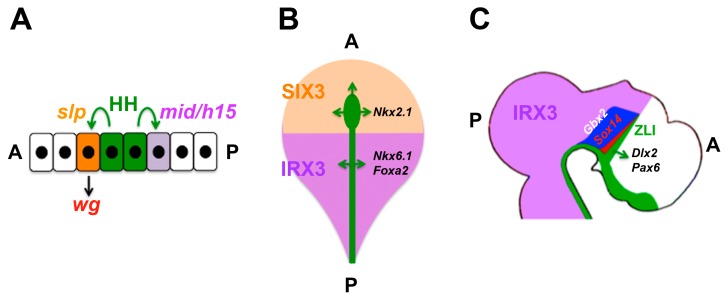
Three examples for domains of differential competence for HH signalling. (**A**) In the *Drosophila* embryo, HH (green) is released at the anterior border of each parasegment [129,130]. Anteriorly, the expression of *slp* (orange) endows cells with competence for the induction of *wg* (red); posteriorly, *mid/h15* (purple) prevents *wg* induction by HH [131,132,133]. (**B**) In the anterior neural plate of the chick embryo, SIX3 (orange) endows cells with competence for the induction of *Nkx1.2* by SHH (green) whereas IRX3 (purple) endows cells with competence for the induction of *NKX6.1* and *FOXA2* posteriorly [141]. (**C**) In the forebrain, SHH (green) induces *Sox14*- and *Gbx2*-positive neurons of the thalamus dose-dependently posterior to the ZLI, but *Dlx2/Pax6*-positive neurons anteriorly [142]. Thalamic competence is mediated by IRX3 posterior to the ZLI [143,148]. A, anterior; P, posterior; ZLI, zona limitans intrathalamica.

Differential spatial competence for HH signalling was first observed in the *Drosophila* embryo where *hh* is expressed in stripes of ectodermal cells at the anterior border of each parasegment. Although the HH response gene *ptc* is induced on either side of these stripes, the HH target *wingless* (*wg*) is induced only anteriorly [129,130]. Subsequently this differential competence was found to be mediated by the differential expression of the transcription factor-encoding genes *sloppy paired* (*slp*) anterior to the *hh* domain and *midline* (*mid*) and *h15* posterior to it. *Slp* and *mid/h15* define the cellular response to HH signalling in their respective domains such that *wg* can only be induced anteriorly. *Slp* and *mid/h15* mutually repress one another, thereby stabilising the respective identities of these competence areas (Figure 4A) [131,132,133].

Differential competence for HH is also observed in the imaginal discs, the epithelial pouches in the *Drosophila* larva that are the progenitors of the adult fly’s appendages some of which are known to be patterned by HH in a morphogen-like fashion [134,135]. In the wing imaginal disc *hh* is expressed in the posterior compartment, and the HH target genes *engrailed* (*en*), *ptc* and *decapentaplegic* (*dpp*) are dose-dependently induced in stripes of different diameters in the anterior compartment. *En* is also expressed in the posterior compartment, however posterior *en* expression does not depend on HH signalling indicating that the GRN downstream of HH differs between both compartments [136]. By contrast, HH synchronises cellular differentiation in the eye imaginal disk [137,138,139]. In the eye-antennal complex, HH promotes cellular proliferation in the dorsal domain, but regulates transcription of *dpp* in the ventral domain [140]. Thus, differential competence for HH is found not only in different imaginal discs (wing—pattering, eye—differentiation), but also in different compartments of one imaginal disc (anterior versus posterior wing disc).

In vertebrates distinct regions in the developing neural tube display differential competence for HH signalling. In the early neural plate of the chick embryo, the homeobox gene *SIX3* is expressed anteriorly in a domain that is complementary to the posterior expression domain of *IRX3*. Both genes encode homeodomain transcription factors that cross-repress each other. Importantly, they define areas of differential competence for two signals: SHH induces *NKX2.1* in the *SIX3* domain, but *NKX6.1* and *FOXA2 (HNF3β)* in the *IRX3* domain; and FGF induces *FOXG1 (BF1)* in the *SIX3* domain and *EN2* in the *IRX3* domain (Figure 4B) [141].

**Figure 5:**
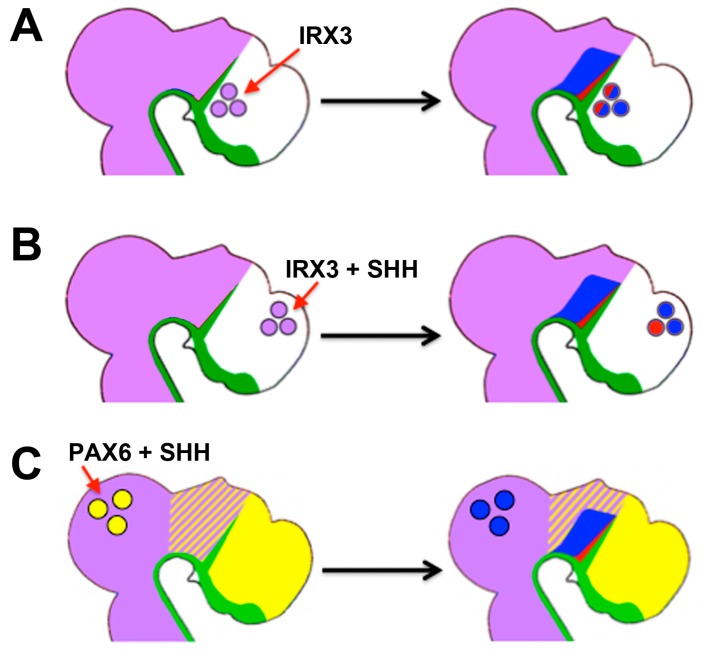
IRX3 and PAX6 endow cells with competence for the induction of thalamic neurons by SHH. (**A**) Ectopic expression of IRX3 (purple) anterior to the ZLI results in a mirror-image duplication of GABAergic (red) and glutamatergic (blue) neurons of the thalamus in the prethalamus. These inductions depend on SHH signalling [143]. (**B**) Ectopic expression of IRX3 in the dorsal telencephalon results in induction of GABAergic and glutamatergic thalamic neurons, but only if the SHH pathway is simultaneously activated. (**C**) Ectopic expression of PAX6 (yellow) and simultaneous activation of the SHH pathway results in induction of thalamic neurons in the dorsal midbrain, suggesting that the overlap of IRX3 and PAX6 defines the competence domain for thalamus formation in response to SHH [148].

Notably, IRX3 continues to function as a mediator of differential competence until later stages of neural development. In the posterior forebrain (diencephalon), a transverse stripe of cells called the zona limitans intrathalamica (ZLI) secretes SHH. In the neuroepithelium posterior to the ZLI, high levels of SHH induce *SOX14*-expressing GABAergic neurons of the thalamus immediately next to the ZLI, whereas lower levels induce *GBX2*-expressing glutamatergic neurons at some distance. In the neuroepithelium anterior to the ZLI (the so-called prethalamus), SHH induces the expression of other factors such as *DLX2* [142,143,144,145,146,147]. These observations indicate that the ZLI is positioned at the border between two different areas of competence for SHH, and gain- and loss-of-function experiments in chick demonstrated that *IRX3*—which is expressed in the thalamus, but not in the prethalamus—mediates this differential competence (Figure 4C). Ectopic expression of *IRX3* in normally *IRX3*-negative regions of the neural tube (prethalamus and telencephalon) endowed cells in these areas with competence to express markers of thalamic differentiation in response to SHH (Figure 5A,B) [143,148]. Although *PAX6* is downregulated by SHH posterior to the ZLI at later stages of thalamic development, the overlap between *PAX6* and *IRX3* expression at earlier stages (before ZLI formation) outlines the area that will eventually form the thalamus. Notably, forced expression of *PAX6* in the normally *PAX6*-negative (but *IRX3*-positive) midbrain endowed cells in this area with thalamic competence. Thus, it appears that both *PAX6* and *IRX3* set up the initial area of competence for thalamus formation (Figure 5C) [148].

A recent study in amphibian embryos demonstrated that *irx3* is expressed in an area that includes the presumptive ZLI whereas its close relatives *irx1* and *irx2* are expressed only in the presumptive thalamus posterior to it. The combination of *irx3, barhl2* and *otx2* (both also encoding homeodomain transcription factors) endows ZLI cells with the competence to express *shh*. In contrast, *irx1/2/3/barhl2/otx2*-expressing cells in the presumptive thalamus lack this competence, suggesting that an ‘IRX code’ regulates a pattern of differential competence in this area and that *irx1* and *irx2* suppress ZLI formation [149].

*IRX3* is also expressed in the spinal cord as part of the homeodomain transcription factor code that mediates the specification of different cell fates along the dorsoventral axis (see above) [150]. In the developing limbs of the mouse embryo, both *Irx3* and *Irx5* are expressed in an anteroposteriorly declining gradient, and genetic loss-of-function experiments have revealed that the combinatorial activity of these two factors antagonises SHH activity from the posterior side of the limb bud: *Irx3/5* double mutant mice display anterior defects such as a lack of femur, tibia and digit 1 in the hindlimb [151]. Whether IRXs are also involved in modulating the competence of cells to respond to SHH in these two systems remains to be established.

The *Drosophila* orthologues of the vertebrate *IRX*s also function as competence factors during the development of the fly’s eye, head, notum and mesoderm [152,153,154,155,156,157]. *IRX2* is expressed in the chick hindbrain, but not in the midbrain, and it endows cells with competence to form a cerebellum in response to FGF signalling from the midbrain-hindbrain boundary—a setup highly reminiscent of the role of *IRX3* at the ZLI. IRX2’s activity is modulated by phosphorylation through the Mitogen Activated Protein Kinase (MAPK) pathway which is activated by FGF signalling: MAPK switches IRX2 from a transcriptional repressor to an activator; thus, the signalling factor itself enhances the competence of its target cells in a positive feedback loop [158]. The *Drosophila* IRXs Araucan and Caupolican also possess putative MAPK phosphorylation sites and may be regulated through this pathway [159,160]. It will be interesting to see whether IRX3’s activity as a competence factor is modulated in a similar fashion by post-translational modifications.

The formation of forelimb-specific versus hindlimb-specific digits in response to SHH signalling provides another example for differential competence within a given tissue type. Several TFs were found to be specifically expressed either in the forelimb (TBX5) or hindlimb bud (TBX4, PITX1) of which *PITX1* appears to function as a selector gene for hindlimb identity in both chick and mouse [161,162]. It remains to be established whether PITX1 functions by endowing cells with hindlimb-like competence for SHH, or whether it has SHH-independent activities in the hindlimb bud.

How are domains of differential competence established? *IRX3* is a target of the WNT signalling pathway which is known to impose posterior identity at the earliest stages of neural development [163,164,165,166]. Thus, competence domains are set up early, before signalling centres such as the midbrain-hindbrain boundary and the ZLI are established, and factors such as IRX2 and IRX3 are therefore often referred to as *pre-patterning factors*. One of the questions that the idea of differential competence domains raises is to what extent signalling centres function instructively (by actively assigning different cell fates), and to what extent they function permissively (by simply revealing an underlying pattern that has been laid down previously). For example, in the case of the ZLI, the dose-dependent induction of GABAergic and glutamatergic thalamic progenitors is instructive whereas the induction of prethalamic neural markers anterior to, and of thalamic neural markers posterior to the ZLI is permissive.

## 5. Conclusions and Outlook

Differential cellular responses to a given signalling factor can be mediated by dose-dependent effects and by the competence of the responding cells. Dose-dependent effects of SHH signalling have been described in the developing spinal cord, limb bud and thalamus. In this review, we have focused on differential responses of cells to HH signalling that are caused by differential competence.

Temporal changes in competence for HHs are observed in several tissues: in the embryonic spinal cord, forebrain and limb bud, SHH initially induces different cell fates in a dose-dependent fashion, but promotes growth at later stages. In the spinal cord, this ‘uncoupling’ of cell fate from SHH is achieved via the induction of a GRN. Part of this GRN are negative feedback loops in the SHH signalling cascade that decrease the responsiveness of cells to the signal (temporal adaptation). The GRNs that mediate HH signalling in other tissues such as the forebrain and limb bud, and whether those involve similar feedback loops, remain to be established. A later switch in competence from patterning and proliferation to axon guidance appears to be mediated by the upregulation of alternative HH receptors in the context of downregulation of the canonical receptors PTC and SMO [121,122,123,124]. Thus, a more systematic analysis of the expression levels of HH pathway components in tissues with different competences for this signal is required. Temporal competence changes can also be mediated by the interaction with other signalling pathways: Notch signalling sensitises neural progenitors to HHs by directly influencing the subcellular distribution of the HH receptor SMO [118,119], and FGF signalling establishes a domain of competence for SHH-mediated floor plate induction at the elongating posterior end of the embryonic axis [112].

Different types of tissue display differential competence for HH signalling. For example, neuron-specific TFs are induced by SHH in the embryonic neural tube (ectoderm) whereas different sets of target genes are activated in the mesoderm of the limb bud. Given that germ layer specification is the earliest and arguably most fundamental step in the diversification of cell fates, it is rather surprising that the expression of a single TF (of the SOXB1 group) is sufficient to endow mesodermal cells in the limb bud with neural competence for SHH and other signals [128]. No equivalent factors have been identified yet that can endow cells elsewhere with mesodermal or endodermal-like competence. Factors such as the SOXB1s will undoubtedly attract great interest, as they are likely to provide useful tools for the reprogramming of stem cells.

Domains of differential competence for HHs are also observed within tissues of the same type such as the imaginal discs of the *Drosophila* embryo or the vertebrate neural tube. A number of transcription factors have been identified that mediate such regional differences in competence (IRX1/2/3, NKX1.2, PAX6, SIX3, etc.) and they are often referred to as pre-patterning factors as they set up regional identity in response to earlier patterning factors. Of course, every factor that is induced as a result of a patterning event—including those induced by HHs—may act as a competence factor for later signals, suggesting a complex spatiotemporal hierarchy of inductive events and their effects, somewhat reminiscent of a Russian doll. Systems biology approaches may help to shed light on this nested character of cell fate assignment.

Until very recently, studies on competence focused on temporal or spatial differences within a given species. A study published earlier this year revealed that chick and zebra finch neural tube tissue possess differential competence for SHH signalling and that this differential responsiveness is essential for the scaling of the dorsoventral pattern of the spinal cord between the larger chick and the smaller finch embryo. In this system, differential competence is caused by different levels of the repressor GLI3: in the finch neural tube GLI3 levels are significantly lower, making this tissue more sensitive to SHH and resulting in a shorter patterning phase [167]. Thus, research on competence is now also impacting on the field of comparative embryology.

Taken together, morphogens such as the HHs are key factors that regulate development by assigning cell fates, and by driving and coordinating proliferation and differentiation. However, the competence for these morphogens constantly changes during this process, providing a four-dimensional framework that is essential to translate signals into meaningful responses and, ultimately, into functional anatomy. Although we have a good appreciation of the molecular mechanisms that underlie HH signalling, and although we know of many different roles that this signalling pathway plays in different embryonic and adult tissues, our understanding of what mediates the competence of cells that respond to HH is in its infancy. Changes of the GRN downstream of HH, alterations of the signal transduction pathway and the input from other signalling pathways may all contribute to transitions of competence. Posttranscriptional, posttranslational and epigenetic mechanisms that could influence cellular competence have not yet received much attention from researchers in the field. Clearly much more work is needed to characterise the relative input of these different mechanisms.

A better understanding of what regulates cellular competence will not only help to answer the somewhat academic question how a small number of signalling pathways can generate tremendous cellular diversity, but may also improve our understanding of a broad range of human pathologies. Defects of the HH signalling pathway can cause a large number of congenital and homeostatic disorders—including different types of cancer. Cancers are caused by uncontrolled proliferation of cells, and HHs can promote proliferation depending on the cellular competence. Thus, it is tempting to speculate that a defective molecular switch that increases the competence of cells to respond to HHs by proliferating could cause cancer. A solid grasp of the molecular basis of cellular competence, and how it can be altered, is likely to open up novel avenues for therapeutic intervention.

## Figures and Tables

**Table 1:** Different roles of Hedgehog (HH) signalling discussed in this review.

Tissue	Role	HH Ligand	Differential Competence?	References
Spinal cord	Ventral induction, later: growth, guidance of commissural axons, glia cell production	SHH	Floor plate vs. ventral interneuron induction (FGF-NKX1.2); motor neuron vs. oligodendrocyte induction (progenitor movement); patterning vs. growth (temporal adaptation, Notch signalling); axon guidance (receptor switch—HHIP, BOC, SMO localisation)	[45,48,93,94,96,97,100,101,103,104,105,106,107,111,112,113,116,118,119,121,122,123,124]
Cerebellum	Expansion	SHH		[66,67,68]
Midbrain	Ventral induction (arcs), later: growth of tegmentum and tectum	SHH	Patterning vs. growth	[51,69]
Hypothalamus	Induction, patterning, expansion	SHH	Patterning vs. growth	[50,120]
Diencephalon	Growth, later: thalamus/prethalamus patterning	SHH	Growth/patterning; prethalamus vs. thalamus (PAX6 and IRX3)	[70,142,143,144,145,146,147,148]
Telencephalon	Subpallium induction, later: neocortex expansion	SHH	Patterning vs. growth (*GLI* downregulation by NKX2.1)	[98,102]
Early neural plate	Patterning	SHH	Anterior *NKX2*.1 vs. posterior *FOXA2* induction (SIX3/IRX3)	[141]
CNS	Stem cell maintenance and activation in response to injury	SHH		[77,78,79,81,82]
Limb bud	Anteroposterior patterning, growth	SHH	Forelimb vs. hindlimb (PITX1); patterning vs. growth	[53,99,151,161,162]
Somites	Sclerotome induction	SHH		[56,57,108]
Muscle	Fibre induction	SHH	Slow-twitch vs. fast fibre (progenitor movement)	[109,110]
Pituitary gland	Induction	SHH		[58]
Teeth	Induction	SHH		[63]
Intestinal epithelium	Inhibition of pancreas induction, later: restriction of stem cell population, enterocyte differentiation	IHH, SHH		[59,75]
Bladder epithelium	Regenerative proliferation	SHH		[83]
Skin	Hair follicle development	SHH		[62]
Lingual epithelium	Taste bud induction	SHH		[61]
Germ line	Leydig cell differentiation, germ cell survival	DHH		[64]
Skeleton	Cartilage differentiation	IHH		[65]
*Drosophila* ectoderm	Segmental patterning	HH	Anterior: *wg* induction (*slp* vs. *mid/h15*)	[130,131,132]
*Drosophila* wing imaginal disc	Anteroposterior patterning	HH	Anterior: *ptc/dpp* induction	[136]
*Drosophila* eye imaginal disc	Photoreceptor differentiation	HH		[137,138,139]
